# The Role of Tetraether Lipid Composition in the Adaptation of Thermophilic Archaea to Acidity

**DOI:** 10.3389/fmicb.2013.00062

**Published:** 2013-04-03

**Authors:** Eric S. Boyd, Trinity L. Hamilton, Jinxiang Wang, Liu He, Chuanlun L. Zhang

**Affiliations:** ^1^Department of Chemistry and Biochemistry, Montana State UniversityBozeman, MT, USA; ^2^Department of Marine Sciences, University of GeorgiaAthens, GA, USA; ^3^State Key Laboratory of Marine Geology, School of Earth and Ocean Sciences, Tongji UniversityShanghai, China

**Keywords:** tetraether lipids, Nitrosocaldus, *amoA*, nitrification, crenarchaeol, community ecology, phylogenetic ecology

## Abstract

Diether and tetraether lipids are fundamental components of the archaeal cell membrane. Archaea adjust the degree of tetraether lipid cyclization in order to maintain functional membranes and cellular homeostasis when confronted with pH and/or thermal stress. Thus, the ability to adjust tetraether lipid composition likely represents a critical phenotypic trait that enabled archaeal diversification into environments characterized by extremes in pH and/or temperature. Here we assess the relationship between geochemical variation, core- and polar-isoprenoid glycerol dibiphytanyl glycerol tetraether (C-iGDGT and P-iGDGT, respectively) lipid composition, and archaeal 16S rRNA gene diversity and abundance in 27 geothermal springs in Yellowstone National Park, Wyoming. The composition and abundance of C-iGDGT and P-iGDGT lipids recovered from geothermal ecosystems were distinct from surrounding soils, indicating that they are synthesized endogenously. With the exception of GDGT-0 (no cyclopentyl rings), the abundances of individual C-iGDGT and P-iGDGT lipids were significantly correlated. The abundance of a number of individual tetraether lipids varied positively with the relative abundance of individual 16S rRNA gene sequences, most notably crenarchaeol in both the core and polar GDGT fraction and sequences closely affiliated with *Candidatus* Nitrosocaldus yellowstonii. This finding supports the proposal that crenarchaeol is a biomarker for nitrifying archaea. Variation in the degree of cyclization of C- and P-iGDGT lipids recovered from geothermal mats and sediments could best be explained by variation in spring pH, with lipids from acidic environments tending to have, on average, more internal cyclic rings than those from higher pH ecosystems. Likewise, variation in the phylogenetic composition of archaeal 16S rRNA genes could best be explained by spring pH. In turn, the phylogenetic similarity of archaeal 16S rRNA genes was significantly correlated with the similarity in the composition of C- and P-iGDGT lipids. Taken together, these data suggest that the ability to adjust the composition of GDGT lipid membranes played a central role in the diversification of archaea into or out of environments characterized by extremes of low pH and high temperature.

## Introduction

Archaea inhabit environments characterized by extremes of salt, temperature, and pH. The ecological dominance of archaea in these environments, including environments with characteristics (e.g., combined low pH and high temperature) that preclude bacterial colonization (Inskeep et al., 2010), has been suggested to result from the evolution of phenotypic traits that enable survival under conditions of chronic energy stress (Valentine, 2007). The lipid membrane is fundamental to energy generation and the maintenance of cellular homeostasis, suggesting that survival in these extreme environments requires highly specialized lipid membranes (van de Vossenberg et al., 1998; Macalady et al., 2004; Baker-Austin and Dopson, 2007). Archaea synthesize a variety of diether and tetraether linked membrane lipids (Yamauchi et al., 1993; Macalady et al., 2004; Valentine, 2007). The membrane lipids synthesized by Crenarchaeota, as well as by some Euryarchaeota, are composed of glycerol dibiphytanyl glycerol tetraethers (GDGTs) (De Rosa et al., 1980, 1986; De Rosa and Gambacorta, 1988; Macalady et al., 2004). GDGTs consist of ether-linked C_40_ polyisoprenoid chains with zero to as many as four cyclopentyl rings and zero or one cyclohexyl ring (i.e., crenarchaeol; Damsté et al., 2002) on each chain (Schouten et al., 2000, 2003). The monolayer arrangement and the ether-linked bonding are thought to confer enhanced thermal stability to the lipid membrane (Thompson et al., 1992). In addition, internal cyclopentyl rings are thought to enhance the thermal stability of the GDGT membrane through increased packing density (Gliozzi et al., 1983; Gabriel and Chong, 2000).

In the upper marine water column, marine sediments, and freshwater sediments, the average number of cyclopentyl rings per GDGT correlates with increasing surface water temperature (Schouten et al., 2002, 2003; Powers et al., 2004). In terrestrial systems, environmental parameters other than temperature also appear to influence GDGT composition. For example, the composition of GDGT lipids recovered from terrestrial geothermal springs such as those in Yellowstone National Park (YNP), Wyoming, USA exhibit varying correlations with pH and to a lesser extent temperature (Schouten et al., 2007; Pearson et al., 2008). In contrast, the composition of GDGT lipids recovered from terrestrial geothermal springs in the Great Basin (GB), Nevada, USA, which tend to not vary considerably in pH, were correlated with the concentration of bicarbonate but not temperature (Pearson et al., 2004). More recent studies targeting circumneutral to alkaline Tibetan hot springs, which also tend to not vary considerably in pH, indicated an overarching temperature effect on the distribution of some but not all GDGTs in these geothermal springs (He et al., 2012).

In addition to environmental studies, a survey of the composition of GDGT lipids in membranes of archaeal isolates cultivated at optimal pH and temperatures also suggests an important role for tetraether lipids in the survival of archaea at elevated temperature and acidic pH (Macalady et al., 2004). Experimentally, it was shown that the thermoacidophiles *Thermoplasma acidophilum* (Euryarchaeota), *Sulfolobus solfataricus* (Crenarchaeota), and *Acidilobus sulfurireducens* (Crenarchaeota) respond to increases in incubation temperature by increasing the number of cyclopentyl rings in their GDGT core lipids (De Rosa et al., 1980; Uda et al., 2001; Boyd et al., 2011b). In addition, *A. sulfurireducens* was shown to increase the number of cyclopentyl rings in GDGT core lipids in response to increasing cultivation medium acidity (Boyd et al., 2011b). Thus, the ability for archaea to adjust the composition of their GDGT lipid membranes is a fundamental phenotype that facilitates their survival in a range of habitats.

Targeted 16S rRNA gene and non-targeted metagenomic characterizations of YNP geothermal spring communities also indicate an important role for both pH and temperature in structuring the composition and function of archaeal assemblages (Meyer-Dombard et al., 2005; Spear et al., 2005; Inskeep et al., 2010). These results suggest that archaea have adapted phenotypes that enable ecological success and persistence in these environments. Considering the apparent role that GDGT lipid cyclization has in maintaining functional membranes across gradients of temperature and pH, these observations imply a link between GDGT lipid composition and the diversification of thermophilic archaea. Here, we examine this link by coordinating an assessment of core (C)- and intact polar (P)-GDGT lipids, archaeal 16S rRNA gene abundance and diversity, and geochemistry in 27 geothermal springs in YNP. In addition, we quantify the distribution and abundance of archaeal ammonia monooxygenase genes (*amoA*) since crenarchaeol has been suggested to be a biomarker for nitrifying archaea (de la Torre et al., 2008; Pitcher et al., 2009). A phylogenetic framework is employed to evaluate the links between the diversification of archaea at the level of 16S rRNA gene evolution, C- and P-iGDGT lipid composition, and environmental characteristics. The results of our study demonstrate the utility of integrating molecular genetic approaches with lipid biogeochemistry in generating a better understanding of archaeal ecology and evolution in terrestrial geothermal environments.

## Materials and Methods

### Sample collection and chemical analyses

Water, mat, and/or sediment samples were collected from a total of 27 sites from four geographically distinct areas in YNP in June through August of 2008: Norris Geyser Basin (samples E1 to E11, E31, E32), Heart Lake Geyser Basin (samples E12 to E22), Imperial Geyser (samples E23 to E30), and Nymph Lake (samples E33 to E39) (Table 1). In addition a soil sample was collected from a point adjacent and up the hydrological gradient at each sampling site. Spring temperature and pH were determined using a temperature compensated model 59002-00 Cole-Parmer electrode. An alcohol thermometer was used to confirm temperature. Conductivity was measured using a YSI model 33 S-C-T meter (Yellow Springs Instrument Company, Inc., Yellow Springs, OH, USA). Conductivity values were standardized to a common temperature of 25.0°C as previously described (Hamilton et al., 2011). Concentrations of ferrous iron (Fe^2+^) and total sulfide (S^2−^) were quantified onsite with a Hach DR/2000 spectrophotometer (Hach Company, Loveland, CO, USA) and Hachferrozine pillows and sulfide reagents, respectively. For both Fe^2+^ and S^2−^ determinations, water samples were filtered (0.22 μm) prior to addition of reagents. Water samples were filtered (0.22 μm) and frozen on site for use in determination of aqueous chemistry. Dissolved nitrate (${\text{NO}}_{\text{3}}^{-}$), nitrite (${\text{NO}}_{\text{2}}^{-}$), ammonia (${\text{NH}}_{\text{4}}^{+}$), chloride (Cl^−^), and sulfate (${\text{SO}}_{\text{4}}^{2+}$) were determined using a model MT-3 segmented flow analyzer (SEALQuAAtro, West Sussex, England) calibrated daily with freshly prepared standards.

**Table 1:** **Location and aqueous chemistry associated with geothermal mat and sediment samples**.

ID	GPScoordinates		pH	Temp. (C)	Cond. (μmhos/cm)	Sal. (%)	S^2−^ (μM)	Fe^2+^ (μM)	${\text{NO}}_{2}^{-}$ (nM)	${\text{NO}}_{3}^{-}$ (nM)	${\text{NH}}_{4}^{+}$ (μM)	Cl^−^ (mM)	${\text{SO}}_{4}^{2+}$ (mM)
E1	N 44°43′39.0″	W 110°42′56.3″	3.12	71.0	3500	2.0	5.9	11.2	34.3	470.7	71.9	8.06	1.10
E2	N 44°43′43.1″	W 110°42′45.2″	2.28	83.0	2600	2.4	4.4	10.7	30.0	1208.6	286.0	0.03	5.19
E3	N 44°43′45.4″	W 110°42′44.9″	2.81	58.3	2600	2.0	12.6	2.3	69.3	594.3	51.7	7.92	1.44
E4	N 44°43′56.8″	W 110°42′35.1″	4.27	79.5	3750	2.1	8.9	0.8	109.3	499.3	11.4	9.79	0.98
E5	N 44°43′59.8″	W 110°42′35.2″	2.55	82.0	3600	2.3	16.8	15.6	35.7	505.7	207.6	6.09	3.39
E6	N 44°43′59.2″	W 110°42′34.4″	2.93	75.0	3600	2.3	12.5	2.2	37.9	682.1	202.6	7.10	2.34
E7	N 44°43′58.9″	W 110°42′33.2″	2.61	22.5	3700	2.3	BD	6.8	35.0	912.9	87.8	10.60	3.86
E8	N 44°43′58.0″	W 110°42′34.4″	3.30	70.0	3300	2.1	14.9	4.0	37.1	311.4	103.7	8.07	1.78
E9	N 44°43′40.4″	W 110°42′36.9″	4.45	64.8	3750	2.5	1.1	11.0	27.1	352.1	143.9	8.27	2.46
E10	N 44°43′35.8″	W 110°42′32.9″	7.17	82.4	6000	4.0	1.4	BD	102.9	355.0	18.2	11.15	0.39
E11	N 44°43′35.8″	W 110°42′32.9″	7.46	60.2	3100	3.0	BD	BD	339.3	946.4	13.8	11.75	0.43
E12	N 44°18′17.8″	W 110°31′19.9″	8.49	47.7	1750	1.1	0.1	0.2	870.0	1980.0	8.6	3.79	1.61
E13	N 44°18′18.8″	W 110°31′19.3″	7.32	83.8	2700	1.9	0.6	BD	187.9	205.7	25.8	4.22	1.82
E14	N 44°18′18.8″	W 110°31′19.3″	3.49	64.8	3200	2.0	11.2	16.6	35.2	264.1	88.6	7.51	3.48
E15	N 44°18′14.1″	W 110°31′16.7″	9.30	69.0	3800	2.8	0.4	9.3	863.6	448.6	3.9	6.41	2.62
E17	N 44°18′15.7″	W 110°31′23.7″	9.10	86.7	4600	3.5	18.7	BD	118.6	138.6	5.9	5.27	1.99
E18	N 44°18′15.8″	W 110°31′23.0″	9.45	54.5	3350	2.4	34.1	5.2	116.4	386.4	1.6	6.26	2.48
E19	N 44°18′15.7″	W 110°31′22.2″	9.57	38.0	2650	1.9	2.2	0.1	204.3	516.4	4.1	5.61	2.13
E22	N 44°18′17.2″	W 110°31′23.9″	8.88	67.0	4000	3.1	2.3	0.2	644.3	636.4	4.9	3.31	2.35
E23	N 44°31′54.6″	W 110°52′33.3″	6.69	55.0	1500	0.9	0.1	BD	276.4	432.9	8.2	4.29	1.10
E24	N 44°31′54.6″	W 110°52′33.3″	8.50	52.4	2600	1.8	2.5	BD	155.0	343.6	1.1	3.43	0.20
E26	N 44°31′54.6″	W 110°52′33.3″	5.80	48.0	1000	0.5	4.7	16.4	167.9	282.1	91.0	3.28	0.33
E29	N 44°31′54.6″	W 110°52′33.3″	4.10	81.5	320	0.1	1.9	4.4	122.1	1782.1	111.0	3.89	0.80
E31	N 44°43′35.8″	W 110°42′32.9″	3.01	39.2	2375	1.5	3.8	19.8	67.9	427.1	94.1	6.66	1.95
E32	N 44°43′45.4″	W 110°42′44.9″	2.75	43.1	3175	2.2	BD	8.5	56.4	272.9	46.5	7.23	1.09
E36	N 44°45′06.9″	W 110°43′42.8″	2.06	32.7	2300	2.6	BD	30.1	268.6	BD	26.7	0.37	0.50
E39	N 44°45′06.9″	W 110°43′42.8″	4.95	16.3	590	0.5	1.6	5.3	497.9	21.4	2.4	0.24	0.19

*Abbreviations: ID, Site identifier; Temp, temperature; Cond, Conductivity; Sal, Salinity; BD, below detection*.

*Detection limits: S^2−^ = 150 nM; Fe^2+^ = 160 nM; ${\text{NO}}_{3}^{-}$ = 3 nM*.

### DNA extraction and quantification

Sediments for molecular analyses were collected aseptically using a flame-sterilized spatula, placed in 1.5 mL centrifuge tubes, and immediately flash-frozen in a dry ice-ethanol slurry for transport to the lab, where they were kept at −80°C for use in molecular analyses. DNA extraction and purification was carried out as previously described (Boyd et al., 2007b). DNA was extracted in duplicate from ∼250 mg of wet weight sediment and equal volumes of each duplicate extraction were pooled. Genomic DNA was quantified using the Qubit DNA Assay kit (Molecular Probes) and a Qubit 2.0 Fluorometer (Invitrogen).

### PCR amplification

Sediment extracts were screened for archaeal 16S rRNA genes amplified using primers 344F (5′-ACGGGGYGCAGCAGGCGCGA-3′) and 915R (5′-GTGCTCCCCCGCCAATTCCT-3′) at an annealing temperature of 61°C. Archaeal *amoA* genes were amplified as previously described, using an annealing temperature of 53°C and the following primer set: Arch-amoAF (5′-STAATGGTCTGGCTTAGACG-3′) and Arch-amoAR (5′-GCGGCCATCCATCTGTATGT-3′) (Francis et al., 2005). For each set of primers, ∼10 ng of purified genomic DNA was subjected to PCR amplification in triplicate using the following cycling conditions: initial denaturation at 94°C (4 min), followed by 30 cycles of denaturation at 94°C (1 min), annealing at specified temperature (1 min), primer extension at 72°C (1.5 min), followed by a final extension step at 72°C for 20 min. Reactions contained 2 mM MgCl_2_ (Invitrogen, Carlsbad, CA, USA), 200 μM each deoxynucleotide triphosphate (Eppendorf, Hamburg, Germany), 0.5 μM forward and reverse primer (Integrated DNA Technologies, Coralville, IA, USA), 0.4 mg mL^−1^ molecular-grade bovine serum albumin (Roche, Indianapolis, IN, USA) and 0.25 units Taq DNA polymerase (Invitrogen, Carlsbad, CA, USA) in a final reaction volume of 50 μL. An equal volume of each triplicate reaction was pooled and purified using a QIA quick PCR Purification Kit (Qiagen, Valencia, CA, USA). Archaeal 16S rRNA and archaeal *amoA* were cloned as previously described (Boyd et al., 2007a) for use as standards in Quantitative PCR (qPCR) (See below).

### 16S rRNA gene pyrotag sequencing

Archaeal 16S rRNA genes were sequenced by the Research and Testing Laboratory (Lubbock, TX, USA) using the primers described above. Pyrotag libraries were amplified using a 454 Genome Sequencer FLX System. Post sequencing processing was performed with Mothur (ver. 1.25.1) (Schloss et al., 2009). Raw libraries were trimmed, filtered for quality, length, and ambiguous base calls using Mothur. Unique sequences were aligned to the SILVA archaeal database and sequences that started or ended before empirically determined positions of the alignment were removed. The resulting unique sequences were pre-clustered to remove amplification and sequencing errors. Chimeras were detected using UCHIME (Edgar et al., 2011) and were removed. Operational taxonomic units (OTUs) were assigned at a sequence dissimilarity of 0.03 using the furthest-neighbor method. Sequences were classified using the Bayesian classifier and the RDP database and manually verified with BLASTn (Tables S3 and S4 in Supplementary Material). Sequences representing each unique OTU have been deposited in the NCBI Sequence Reads Archive under the accession number SRR648329. Representative sequences of each OTU are also presented in Table S8 in Supplementary Material.

### Phylogenetic analysis

The phylogenetic position of archaeal 16S rRNA genes was evaluated by approximate likelihood-ratio tests (Anisimova and Gascuel, 2006) as implemented in PhyML-aBayes (version 3.0.1) (Anisimova et al., 2011). The archaeal 16S rRNA gene phylogeny was rooted with 16S rRNA genes from *Clostridium acetobutylicum* ATCC 824 (AE001437) and *Caldicellulosiruptor saccharolyticus* DSM 8903 (CP000679). Phylogenies were constructed using the General Time Reversible (GTR) substitution model with a proportion of invariable sites and gamma-distributed rate variation as recommended by jModeltest (ver. 3.8) (Darriba et al., 2012). The consensus phylogram was rate-smoothed using the multidimensional version of Rambaut’s parameterization as implemented in PAUP (ver. 4.0) (Swofford, 2001). Rate-smoothing was performed according to the parameters identified using jModeltest. This included the identification of the best fit substitution model and specification of the gamma distribution of rate variation across sites, proportion of invariant sites, nucleobase frequencies, and rate matrix for each phylogram.

### Quantitative PCR

Quantitative PCR was used to estimate the number of archaeal 16S rRNA and archaeal *amoA* genes in DNA extracts. Methods for qPCR generally followed those developed by Boyd et al. (2011a). Archaeal 16S rRNA and archaeal *amoA* gene clones, generated as described above, were used in generating standard curves for use in relating template copy number to threshold qPCR amplification signal. Two clones for each target gene were used to generate standard curves. The abundance of 16S rRNA and *amoA* gene clones used in generating the standard curves varied by less than a factor of 1.0 and thus were averaged for use in calculating the average template abundances and SD in template abundances from replicate qPCRs. For quantification of archaeal 16S rRNA genes, a standard curve was generated over five orders of magnitude from 5.6 × 10^2^ to 3.1 × 10^7^ copies of template per assay (R^2^ = 0.995). For quantification of archaeal *amoA* genes, a standard curve was generated over five orders of magnitude from 9.6 × 10^2^ to 2.2 × 10^7^ copies per assay (R^2^ = 0.993). The detection limit for both archaeal 16S and *amoA* was ∼15 and 10 copies per ng of extracted DNA, respectively. qPCR assays were performed in a Rotor-Gene 300 quantitative real-time PCR machine (Qiagen, Valencia, CA, USA) in 0.5 mL optically clear PCR tubes (Qiagen, Valencia, CA, USA) using a SsoFast™ EvaGreen Supermix qPCR Kit (Bio-Rad Laboratories, Hercules, CA, USA). qPCR assays were amended with molecular-grade bovine serum albumin (Roche, Indianapolis, IN, USA) to a final concentration of 0.4 mg mL^−1^. qPCR cycling conditions were as follows: initial denaturation (95°C for 10 min) followed by 40 cycles of denaturation (95°C for 10 s), annealing (55°C for archaeal 16S rRNA genes, or 53°C for *amoA* genes, for 15 s), and extension (72°C for 20 s). Specificity of the qPCR assays was verified by melt curve analysis. Negative control assays were performed in the absence of template DNA. Each assay was performed in triplicate and the reported template abundances are the average and SD of triplicate determinations for the two control plasmids.

### Tetrather lipid detection and quantification

Freeze-dried sediments and mat material were homogenized with a mortar and pestle before lipid extraction. Approximately 5 g of dried solids were extracted following a modified Bligh and Dyer extraction procedure (White et al., 1979). Samples were sequentially extracted six times by sonication for 15 min using a mixture of MeOH, dichloromethane (DCM) and phosphate buffer (pH 7.4) (2:1:0.8, v/v/v; total volume of ∼50 mL). After each sonication, samples were centrifuged at 2500 rpm for 5 min, and were then subjected to the next round of extraction. All extractions were pooled and DCM (1 volume) and phosphate buffer (1 volume) were added to the combined extract (0.9 volume) to achieve phase separation. The bottom DCM phase was collected into a glass tube and dried under N_2_. A known amount of C_46_ GDGT internal standard was added to all samples (Huguet et al., 2006). The total lipid extract was divided into apolar and polar fractions by sequential elution with hexane:DCM (9:1, v/v) and DCM:MeOH (1:1, v/v), respectively. One half of the polar fraction was hydrolyzed according to Wei et al. (2011) and the other half was analyzed directly. The samples were then re-dissolved in hexane:propanol (99:1, v/v), and filtered through 0.45 μm PTFE filters prior to injection on the HPLC-MS.

Glycerol dibiphytanyl glycerol tetraethers were analyzed on an Agilent 1200 liquid chromatography equipped with an automatic injector coupled to QQQ 6460 MS and loaded with Mass Hunter software according to the procedures of  Zhang et al. (2012). Separation of peaks was achieved using a Prevail Cyano column (2.1 mm × 150 mm, 3 μm; Alltech Deerifled, IL, USA) maintained at a temperature of 40°C. Injection volume was 5 μL. Two solvents were used in the elution of GDGTs, solvent A (*n*-hexane) and solvent B (90% *n*-hexane: 10% isopropanol). GDGTs were first eluted isocratically with 90% solvent A and 10% solvent B for 5 min, followed by a linear gradient to 18% solvent B in 45 min. Solvent was held for 10 min in 100% solvent B and was then allowed to re-equilibrate in a mixture of solvents A:B (9:1, v/v) for 10 min. Detection of GDGTs was performed using an Agilent 6460 triple-quadrupole mass spectrometer (MS) with an atmospheric pressure chemical ionization (APCI) ion source. The scanning type used was the single ion monitoring mode of protonated molecules. The conditions for APCI/MS were as follows: nebulizer pressure 40 psi, vaporizer temperature 350°C, drying gas (N_2_) flow 5 L/min and temperature 250°C, capillary voltage 3 kV, and corona 4 μA. All samples were quantified by adding a known amount of an internal C_46_ standard. GDGTs identified by LC-MS are reported according to the nomenclature of Schouten et al. (2003) and as modified in Pearson et al. (2008). Importantly, GDGT-4′ is an isomer of GDGT-4 (four cyclopentyl rings), GDGT-5′ is an isomer of GDGT-5 (five cyclopentyl rings), and neither GDGT-5 nor 5′ is crenarchaeol (four cyclopentyl rings plus one cyclohexyl ring) (Sinninghe Damsté et al., 2002). This conclusion follows from the fact that GDGT-4 and 4′ do not coelute with GDGT-5 and 5′. In contrast, crenarchaeol typically coelutes with GDGT-4 and its abundance can be estimated from linear interpolation of authentic mass spectra of GDGT-4 and crenarchaeol. The weighted average number of GDGT rings per lipid molecule [Ring Index (RI)] was calculated according to the formula: RI = [%GDGT-1 + 2*(%GDGT-2) + 3*(GDGT-3) + 4*(%GDGT-4 + %GDGT-4′) + 5*(%GDGT-5 + %GDGT-5′) + 6*(%GDGT-6) + 7*(%GDGT-7) + 8*(%GDGT-8)]/100 as modified from Schouten et al. (2007). The relative abundance of crenarchaeaol and the isomer of crenarchaeol were not included in this calculation.

### Community ecology and statistical analysis

Phylocom (ver. 4.1) (Webb et al., 2008) was used to construct a community phylogenetic distance matrix using Rao phylogenetic distances derived from the rate-smoothed archaeal 16S rRNA gene cladogram as previously described (Boyd et al., 2010). Euclidean distance matrices derived from the environmental variables listed in Table 1 were constructed using the base package within R (version 2.10.1). Chemical measurements that were below the limit of detection (Table 1 and footnotes) were given the minimal detectable value for the purposes of statistical analysis. In addition, the relative abundance of C- and P-iGDGTs was used to construct Euclidean distance matrices describing the dissimilarity of the lipid profiles between samples. Model selection via Akaike Information Criteria adjusted for small sample size (AICc) and Mantel regressions (999 permutations) were used to examine the extent to which the dissimilarity matrices co-varied. Model Selection and Mantel regressions were performed using the R packages Ecodist (Goslee and Urban, 2007), pgirmess (ver. 1.4.3)^1^, vegan^2^, and labdsv^3^. We considered the model with the lowest AICc value to be the best and evaluated the relative plausibility of each model by examining differences between the AICc value for the best model and values for every other model (ΔAICc) (Burnham and Anderson, 2002; Johnson and Omland, 2004). Models with ΔAICc < 2 were considered strongly supported by the data, and models with ΔAICc > 10 and/or Mantel significance (*p*) values > 0.05 can be considered to have essentially no support from the data. Principle coordinate ordination (PCO) of Rao 16S rRNA gene phylogenetic similarity was performed using the R package vegan (see text footnote 2). Pearson linear regression analysis was used to examine relationships between gene abundances, geochemistry, individual C- and P-iGDGTs, C- and P-iGDGT ring indices, and archaeal 16S OTUs. Regression analysis was performed using the program XL Stat (ver. 2008.7.03).

## Results

### Sample site description

The temperature of the hot spring water where samples were collected ranged from 16.3 to 86.7°C and pH from 2.06 to 9.57 (Table 1). A number of the chemical analytes co-varied (Table S1 in Supplementary Material), with pH varying inversely with Fe^2+^ (Pearson *R* = −0.58, *p* < 0.01) and ${\text{NH}}_{4}^{+}$ (Pearson *R* = −0.67, *p* < 0.01) and positively with ${\text{NO}}_{2}^{-}$ (Pearson *R* = 0.61, *p* < 0.01). Concentrations of ${\text{NO}}_{2}^{-}$ and ${\text{NH}}_{4}^{+}$ were inversely correlated (Pearson *R* = −0.51, *p* < 0.01).

### Source of hot spring C- and P-iGDGT lipids

**Figure 1:**
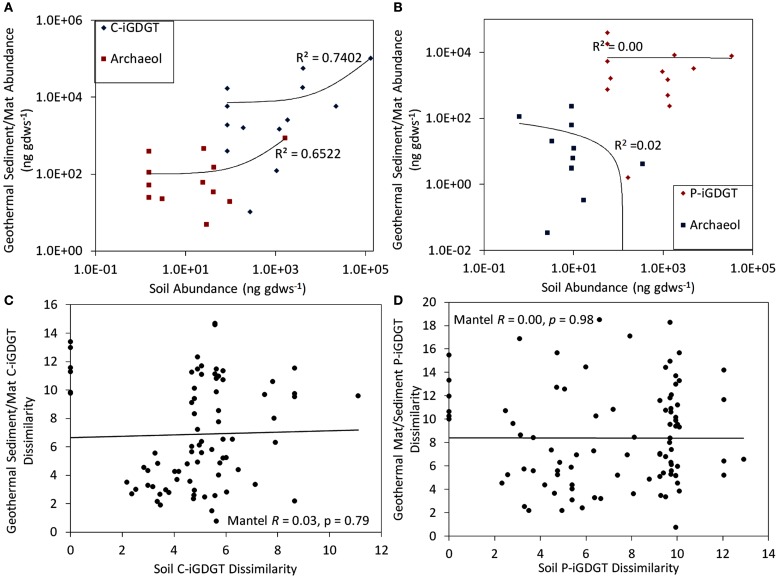
**Relationship between the abundance of C-iGDGT and C-archaeol in 26 geothermal sediments/mats plotted as a function of the abundance of the C-iGDGT and C-archaeol in surrounding soils (A)**. Relationship between the abundance of P-iGDGT and P-archaeol in 26 geothermal sediments/mats plotted as a function of the abundance of the P-iGDGT and P-archaeol in surrounding soils **(B)**. Mantel regression between a matrix describing the dissimilarity in the composition of C-iGDGTs in 26 geothermal sediments/mats plotted as a function of the dissimilarity in the composition of C-iGDGTs in surrounding soils **(C)**. Mantel regression between a matrix describing the dissimilarity in the composition of P-iGDGTs in 26 geothermal sediments/mats plotted as a function of the dissimilarity in the composition of P-iGDGTs in surrounding soils **(D)**. Data in panel A and B are plotted on a log–log scale with a linear regression depicting the relationship. The data used to construct plots **(A–D)** is presented in Tables 2 and 3.

C- and P-archaeol and iGDGTs were recovered from 25 of the 27 springs analyzed (exceptions are E13 and E23) (Tables 2 and 3) and their abundance and structural composition (C- and P-iGDGTs only) were compared to those from surrounding terrestrial soils (Table S2 in Supplementary Material) in order to establish the potential for exogenous input into the system. The abundance of C-archaeol and C-iGDGTs tended to be greater in hot spring sediments/mats than in surrounding soils; however, the abundance of these compounds in hot spring sediments/mats and soils was correlated (Pearson *R*^2^ = 0.65 and 0.74, respectively) (Figure 1A). In contrast, the abundance of P-archaeol and P-iGDGTs in hot spring sediments/mats and in surrounding soils were not correlated (Pearson *R*^2^ = 0.00 and 0.02, respectively) (Figure 1B). To compare the structural composition of C- and P-iGDGTs in hot spring sediments/mats with those obtained from surrounding soils, matrices describing the dissimilarity in the relative abundance of individual lipid structures (i.e., GDGT-0 to GDGT-8) were constructed and subjected to Mantel regression. Comparison of the compositions of C-iGDGT and P-iGDGTs obtained from both hot spring sediment/mat and surrounding soils revealed statistically insignificant relationships (Mantel *R* = 0.00 and 0.03, respectively; *p*-values = 0.79 and 0.98, respectively) (Figures 1C,D), indicating that the lipid structures identified in the hot spring sediment/mats are not likely to be the result of exogenous input.

**Table 2:** **Relative abundance of C-iGDGT lipids in geothermal mat and sediment samples**.

ID	C-iGDGT*														Ring index	C-iGDGT (ng/gdws)	C-Archaeol (ng/gdws)
	0	0′	1	2	3	4	4′	5	5′	Cren	Cren. Iso	6	7	8	
E1	0.2	1.3	2.6	9.6	21.6	48.2	0.4	10.8	0.0	0.5	0.1	4.7	0.1	0.1	3.7	15162.6	135.6
E2	0.0	1.1	1.5	3.7	10.4	55.6	0.8	19.8	0.0	0.0	0.0	7.2	0.0	0.0	4.1	10.5	0.1
E3	0.3	2.6	1.6	8.7	8.2	58.6	0.6	13.4	0.1	0.6	0.0	5.3	0.1	0.1	3.8	1601.4	22.7
E4	0.1	0.6	1.0	3.0	9.7	75.7	0.3	6.8	0.1	0.6	0.0	2.2	0.0	0.0	3.9	5792.0	18.9
E5	0.1	1.6	2.8	7.5	16.5	48.4	0.8	14.9	0.1	0.3	0.0	6.7	0.1	0.1	3.8	56527.7	452.1
E6	0.2	1.2	2.0	5.8	13.9	44.0	1.8	21.4	0.4	0.3	0.0	8.8	0.1	0.1	4.0	104666.1	851.2
E7	0.3	2.4	3.2	6.6	7.8	54.9	0.6	11.9	0.2	0.6	0.2	10.7	0.2	0.3	3.9	1507.2	34.4
E8	0.2	1.6	2.6	7.7	16.8	54.9	0.3	10.5	0.1	0.4	0.0	4.5	0.2	0.2	3.7	18178.5	148.1
E9	0.5	4.4	2.0	10.5	12.2	42.8	1.1	13.6	0.3	0.4	0.0	12.3	0.0	0.0	3.8	122.5	4.9
E10	0.5	12.1	12.1	16.2	25.3	27.2	0.2	3.6	0.0	1.3	0.1	1.4	0.0	0.0	2.6	118.3	2.2
E11	3.3	10.9	8.2	16.0	17.2	32.0	0.2	4.7	0.2	4.2	0.2	2.9	0.0	0.0	2.8	164.5	5.4
E12	0.5	15.9	22.2	22.2	17.7	13.2	0.3	2.7	0.0	2.7	0.3	2.3	0.0	0.0	2.2	6601.6	87.0
E14	0.5	2.3	2.0	5.2	12.0	40.7	1.6	22.0	0.2	0.5	0.1	12.7	0.0	0.0	4.1	2558.9	60.1
E15	0.7	14.5	10.6	24.6	15.4	19.3	0.4	4.0	0.0	8.2	1.0	1.3	0.0	0.0	2.6	169.2	10.6
E17	0.7	9.8	10.9	17.5	24.7	29.1	0.3	4.2	0.0	1.2	0.2	1.4	0.0	0.0	2.7	338.7	22.5
E18	0.2	6.3	10.2	23.0	37.3	22.1	0.0	0.3	0.0	0.6	0.0	0.1	0.0	0.0	2.6	850.7	4.7
E19	0.3	7.2	10.9	20.0	32.0	27.2	0.1	1.1	0.0	0.8	0.0	0.4	0.0	0.0	2.7	8151.8	76.6
E22	1.5	7.9	11.0	18.3	29.8	29.4	0.1	0.3	0.0	1.5	0.1	0.1	0.0	0.0	2.7	17062.9	392.9
E24	0.5	3.3	2.9	4.8	14.2	72.4	0.0	0.4	0.0	1.2	0.2	0.1	0.0	0.0	3.5	5808.2	111.6
E26	0.7	36.5	7.0	7.8	10.8	35.3	0.1	0.5	0.0	0.9	0.1	0.2	0.0	0.0	2.1	1912.6	50.8
E29	1.0	2.1	3.1	4.9	11.9	39.0	1.2	21.6	0.1	0.5	0.0	14.6	0.0	0.0	4.1	398.2	24.3
E31	0.2	2.0	3.3	8.2	17.1	53.4	0.4	8.5	0.1	0.5	0.1	6.0	0.1	0.1	3.7	482.8	5.5
E32	1.1	6.3	5.4	12.1	12.9	47.3	0.5	9.9	0.2	0.5	0.1	3.7	0.0	0.0	3.4	132.3	5.2
E36	0.3	3.4	2.3	6.4	13.2	53.0	0.5	11.0	0.1	0.6	0.1	9.0	0.1	0.1	3.8	642.6	12.2
E39	0.7	9.2	9.7	19.6	25.6	26.2	0.3	4.9	0.0	1.5	0.1	2.1	0.0	0.0	2.8	497.0	68.1

*m/z ratios for each GDGT (left to right) are as defined by (Schouten et al., 2003; Pearson et al., 2008).

**Table 3:** **Relative abundance of P-iGDGTs in geothermal mat and sediment samples**.

ID	P-iGDGT*														Ring index	P-iGDGT (ng/gdws)	P-Archaeol (ng/gdws)
	0	0′	1	2	3	4	4′	5	5′	Cren.	Cren. Iso.	6	7	8	
E1	0.2	2.0	3.8	10.0	17.1	37.9	1.9	15.5	0.7	2.9	0.9	6.4	0.5	0.3	3.8	1542.7	1.0
E2	0.0	3.0	0.1	4.3	9.7	57.6	9.4	10.1	0.0	0.0	0.0	5.8	0.0	0.0	3.9	1.6	0.0
E3	0.2	1.7	1.4	6.5	15.7	59.8	0.6	9.5	0.1	0.4	0.0	3.2	0.7	0.2	3.8	1633.6	20.4
E4	0.1	1.1	2.2	6.1	18.4	52.9	2.3	12.2	0.5	0.2	0.0	3.9	0.2	0.1	3.8	495.5	0.0
E5	0.5	4.5	4.6	16.2	18.0	30.2	6.8	9.2	3.1	0.2	0.0	5.7	0.6	0.5	3.4	3282.4	0.0
E6	0.7	1.5	2.8	6.8	5.1	66.9	5.7	3.6	1.5	1.3	0.0	3.7	0.2	0.2	3.8	7786.3	4.1
E7	0.1	2.6	3.2	11.4	23.6	42.9	0.7	7.9	0.1	0.8	0.0	6.2	0.3	0.2	3.6	1494.9	6.2
E8	0.3	3.5	5.4	14.3	23.6	35.0	1.8	9.5	0.6	0.4	0.0	4.8	0.5	0.4	3.4	2587.5	0.3
E9	0.2	4.8	2.6	13.8	17.1	45.0	1.2	10.0	0.2	0.5	0.0	4.6	0.0	0.0	3.5	233.2	12.1
E10	0.0	11.9	13.4	19.1	23.4	19.9	0.3	0.5	0.0	10.8	0.3	0.2	0.0	0.0	2.6	192.2	0.0
E11	0.5	13.8	8.9	21.3	13.2	12.7	0.3	1.0	0.2	26.9	0.6	0.7	0.0	0.0	2.9	748.4	9.6
E12	0.1	17.3	27.1	25.1	19.7	7.1	0.1	0.5	0.0	2.3	0.2	0.3	0.0	0.0	1.8	34837.8	73.3
E14	0.2	2.5	2.0	5.6	13.4	39.3	2.1	23.2	0.2	0.5	0.1	10.9	0.0	0.0	4.0	8280.2	111.8
E15	0.1	12.4	10.6	40.2	17.1	9.6	0.5	0.9	0.0	7.6	0.7	0.2	0.0	0.0	2.3	890.7	3.6
E17	0.2	9.3	13.1	26.8	28.0	20.2	0.3	0.0	0.0	1.8	0.2	0.0	0.0	0.0	2.4	1056.9	7.4
E18	0.0	5.1	8.6	22.8	37.2	24.9	0.0	0.3	0.0	1.0	0.1	0.1	0.0	0.0	2.7	850.1	1.0
E19	0.1	4.9	9.8	21.4	35.4	26.8	0.1	0.4	0.0	1.1	0.1	0.1	0.0	0.0	2.7	25532.4	29.4
E22	0.4	6.8	11.8	19.9	30.2	28.0	0.2	0.4	0.0	2.1	0.2	0.1	0.0	0.0	2.7	39486.4	61.0
E24	0.1	2.7	2.9	6.1	17.2	67.5	0.0	0.6	0.0	2.5	0.2	0.1	0.0	0.0	3.5	17832.9	3.1
E26	0.8	40.2	10.3	10.7	10.9	24.7	0.3	0.7	0.1	0.8	0.3	0.1	0.0	0.0	1.7	5309.2	226.1
E29	0.1	1.8	2.5	4.2	12.9	40.6	1.7	22.7	0.3	0.5	0.0	12.7	0.0	0.0	4.1	741.8	3.0
E31	0.2	3.4	3.6	9.9	17.4	53.1	0.6	6.6	0.1	1.0	0.0	4.0	0.1	0.1	3.5	333.0	14.7
E32	0.2	11.5	10.1	21.9	18.5	34.5	0.3	2.0	0.0	0.6	0.0	0.4	0.0	0.0	2.6	524.3	20.4
E36	0.2	5.7	3.2	8.6	14.8	47.0	1.0	10.2	0.2	0.8	1.5	6.6	0.1	0.1	3.6	445.5	2.1
E39	0.0	4.3	8.8	24.9	40.3	20.4	0.1	0.3	0.0	0.7	0.1	0.0	0.0	0.0	2.7	3439.5	0.0

*m/z ratios for each GDGT (left to right) are as defined by (Schouten et al., 2003; Pearson et al., 2008).

### Geochemical controls on C-iGDGT composition

A matrix that describes the dissimilarity in the composition of C-iGDGTs could be best explained by variation in environmental pH (ΔAICc = 0.00, Mantel *R*^2^ = 0.36, *p* < 0.01) (Table 4). Models describing the dissimilarity in the concentrations of ${\text{NO}}_{2}^{-}$ (ΔAICc = 95.9, Mantel *R*^2^ = 0.14, *p* < 0.01) and ${\text{NH}}_{4}^{+}$ (ΔAICc = 132.5, Mantel *R*^2^ = 0.04, *p* = 0.01) were also significant predictors of the dissimilarity in the composition of C-iGDGTs. However, given that ${\text{NO}}_{2}^{-}$ and ${\text{NH}}_{4}^{+}$ both co-vary with pH, it is unclear if these parameters represent individual controls on C-iGDGT composition. The RI, which describes the average number of rings per GDGT, was inversely correlated with pH (Pearson *R* = −0.82, *p* < 0.01) (Figure 3A; Table S3 in Supplementary Material). The RI was not significantly correlated with temperature (Figure 3B; Table S3 in Supplementary Material). As expected, the relative abundance of a number of individual C-iGDGTs with few rings (e.g., GDGT-0 to GDGT-3) exhibited significant and positive relationships with pH indicating they are more prevalent in alkaline environments. In contrast, the relative abundance of individual C-iGDGTs with a greater number of rings (e.g., GDGT-4 to GDGT-8) exhibited significant and inverse relationships with pH indicating their prevalence in acidic environments. The relative abundance of crenarchaeol and its isomer in the C-iGDGT fraction, both of which contain four cyclopentyl and one cyclohexyl rings, were positively correlated with pH.

**Table 4:** **Model ranking using ΔAICc and Mantel correlation coefficients (*R*^2^) where C-iGDGT or P-iGDGT distance is the response variable**.

C-iGDGT				P-iGDGT			
Model	ΔAICc	Mantel *R*^2^	*P*-value	Model	ΔAICc	Mantel *R*^2^	*P*-value
pH	0.0	0.36	<0.01	pH	0.0	0.17	<0.01
${\text{NO}}_{2}^{-}$	95.9	0.14	<0.01	${\text{NO}}_{2}^{-}$	35.7	0.08	<0.01
${\text{NH}}_{4}^{+}$	132.5	0.04	0.01	${\text{NH}}_{4}^{+}$	49.0	0.04	0.01
Salinity	141.0	0.01	0.18	Salinity	55.2	0.02	0.08
Temperature	144.2	0.00	0.27	Temperature	60.8	0.00	0.28
Conductivity	144.3	0.00	0.41	Conductivity	61.2	0.00	0.42
S^2−^	145.2	0.00	0.68	${\text{NO}}_{3}^{-}$	61.2	0.00	0.47
Fe^2+^	145.2	0.00	0.66	Cl^−^	61.8	0.00	0.59
${\text{NO}}_{3}^{-}$	145.3	0.00	0.71	${\text{SO}}_{4}^{2+}$	61.9	0.00	0.61
${\text{SO}}_{4}^{2+}$	145.6	0.00	0.94	S^2−^	62.1	0.00	0.71
Cl^−^	145.6	0.00	1.00	Fe^2+^	62.4	0.00	0.89

*Explanatory variables of each model are associated with the criteria by which they were ranked*.

### Geochemical controls on P-iGDGT composition

The abundance of total P-iGDGTs was positively correlated with pH (Pearson *R* = 0.46, *p* < 0.01) (Table S4 in Supplementary Material). A matrix that describes the dissimilarity in the composition of P-iGDGTs could be best explained by variation in environmental pH (ΔAICc = 0.00, Mantel *R*^2^ = 0.17, *p* < 0.01) (Table 4). The RI was inversely correlated with pH (Pearson *R* = −0.71, *p* < 0.01) (Figure 3A; Table S4 in Supplementary Material). The P-iGDGT RI was not correlated with temperature (Figure 3B and Table S4 in Supplementary Material). Similar to the C-iGDGT fraction, the relative abundance of a number of individual P-iGDGTs with fewer rings (e.g., GDGT-0 to GDGT-3) exhibited significant and positive relationships with pH indicating they are more prevalent in alkaline environments. In contrast, the relative abundance of individual P-iGDGTs with a greater number of rings (e.g., GDGT-4 to GDGT-8) exhibited a significant inverse relationship with pH indicating their prevalence in acidic environments. The relative abundance of crenarchaeol and its isomer in the P-iGDGT fraction, both of which contain four cyclopentyl and one cyclohexyl rings, were positively correlated with pH although neither relationship was statistically significant.

### Comparison of hot spring C- and P-archaeal lipids (GDGTs and archaeol)

**Figure 2:**
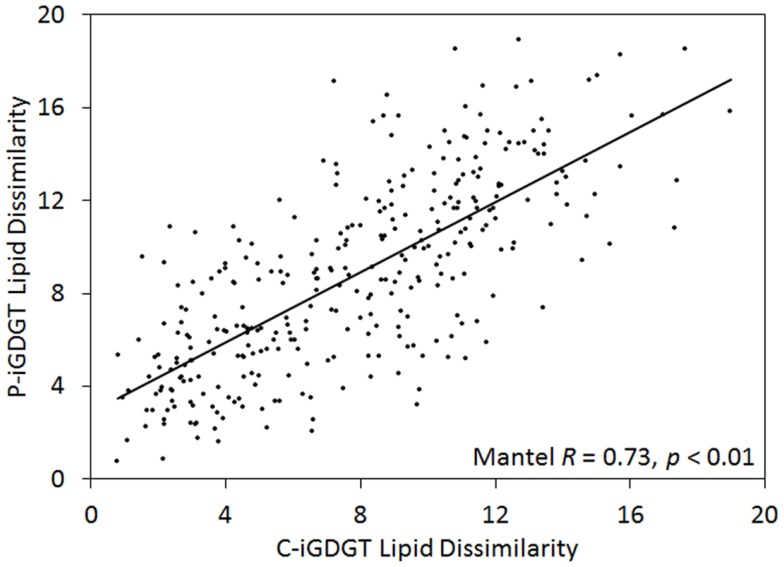
**Mantel regression of matrices describing the dissimilarity in the composition of 26 geothermal spring sediment/mat C-iGDGTs and P-iGDGTs**. The relative abundances of individual lipids used to construct the matrices are presented in Tables 2 and 3.

The abundance of C-iGDGT and P-iGDGT varied in the springs analyzed (Tables 2 and 3, respectively) but were not significantly correlated to each other (Pearson *R* = 0.13, *p* = 0.51) (Table 5). Likewise, the amount of archaeol in the core and polar fractions varied in the springs analyzed (Tables 2 and 3, respectively) but were not significantly correlated to each other (Pearson *R* = −0.03, *p* = 0.88) (Table 5). However, the ratio of the abundance of P-iGDGT to C-iGDGT (i.e., total P-iGDGT/total C-iGDGT) correlated inversely with pH (Pearson *R* = −0.47, *p* = 0.02) and positively with temperature (Pearson *R* = 0.47, *p* = 0.02) (data not shown), suggesting that a combination of high temperature and acidic pH may favor rapid turnover or degradation of C-iGDGT or enhanced synthesis of P-iGDGTs. Matrices describing the dissimilarity in the composition of C- and P-iGDGTs were significantly correlated (Mantel *R* = 0.73, *p* < 0.01) (Figure 2). As such, the relative abundance of individual C- and P-iGDGT lipids were generally significantly correlated (*p* < 0.05) with the sole exception being the relative abundance of GDGT-0 which was not significantly correlated in the C- and P-iGDGT fractions (Pearson *R* = 0.27, *p* = 0.17) (Table 5).

**Table 5:** **Pearson correlation between the relative abundance of GDGT and archaeol lipids in the C- and P-fractions in 26 geothermal spring samples**.

Lipid	Pearson *R*	*p*-value
GTGT-0 (1304)	0.27	0.17
GDGT-0 (1302)	0.97	< 0.01
GDGT-1 (1300)	0.97	< 0.01
GDGT-2 (1298)	0.92	< 0.01
GDGT-3 (1296)	0.79	< 0.01
GDGT-4′ (1294)	0.84	< 0.01
GDGT-4 (1294)	0.55	0.00
GDGT-5 (1292)	0.80	< 0.01
GDGT-5′ (1292)	0.44	0.02
Cren. (1292)	0.57	0.00
Cren. iso. (1292)	0.40	0.04
GDGT-6 (1290)	0.95	< 0.01
GDGT-7 (1288)	0.83	< 0.01
GDGT-8 (1286)	0.68	< 0.01
Total GDGT	0.13	0.51
Total Archaeol	−0.03	0.88

**Figure 3:**
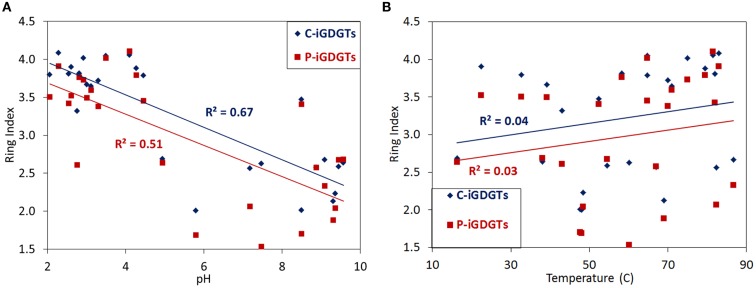
**C-iGDGT and P-iGDGT ring indices plotted as a function of spring pH (A) and spring temperature (B)**.

### Abundance of archaeal 16S rRNA and *amoA* genes

Archaeal 16S rRNA genes were detected in 26 of the 27 springs analyzed (Table S5 in Supplementary Material). In geothermal mat/sediment extracts containing archaeal 16S rRNA genes, the abundance ranged from 1.4 × 10^3^ to 1.6 × 10^8^ templates/ng of DNA extracted. Variation in the abundance of archaeal 16S rRNA genes was not significantly correlated with any of the measured geochemical parameters and could best be described by variation in salinity (Pearson *R* = −0.31, *p* = 0.08) (Table S6 in Supplementary Material). Archaeal *amoA* genes were detected in 16 of the 27 environments examined (Table S5 in Supplementary Material). In geothermal mat/sediment extracts containing archaeal *amoA* genes, the abundance ranged from 1.2 × 10^1^ to 5.2 × 10^4^ copies/ng of DNA extracted. The abundance of archaeal *amoA* was significantly and positively correlated with the concentration of ${\text{NO}}_{2}^{-}$ (Pearson *R* = 0.45, *p* = 0.02) and ${\text{NO}}_{3}^{-}$ (Pearson *R* = 0.57, *p* < 0.01) (Table S6 in Supplementary Material).

The abundance of archaeal 16S rRNA genes was not significantly correlated with the absolute abundance (i.e., relative abundance of individual C-iGDGT lipids multiplied by the total C-iGDGT detected) of any individual C-iGDGT lipid, total C-iGDGT lipids, or C-archaeol (Table S7 in Supplementary Material). In contrast, the abundance of archaeal *amoA* genes was strongly correlated with the abundance of C-crenarchaeol (Pearson *R* = 0.34, *p* = 0.09) and significantly and positively correlated with the abundance of its isomer (Pearson *R* = 0.55, *p* < 0.01). In addition, the abundance of archaeal *amoA* genes was significantly and positively correlated with the absolute abundance of C-iGDGT-0 and C-iGDGT-1 (Pearson *R* = 0.42 and 0.40, *p* = 0.03 and 0.04, respectively).

The abundance of archaeal 16S rRNA genes was not significantly correlated with the abundance of total P-iGDGT lipids or P-archaeol, but was significantly correlated with the absolute abundance of P-iGDGT-5 (m/z = 1292) and P-iGDGT-6 (m/z = 1290) (Pearson *R* = 0.67 and 0.65, *p* < 0.01 and < 0.01, respectively) (Table S8 in Supplementary Material). The abundance of archaeal *amoA* genes was significantly and positively correlated with abundance of total P-iGDGTs (Pearson *R* = 0.56, *p* < 0.01) and the absolute abundance of P-iGDGT-1, P-iGDGT-2, and P-iGDGT-3. Intriguingly, the abundance of archaeal *amoA* genes was also significantly correlated with the abundance of P-crenarchaeol and its isomer (Pearson *R* = 0.59 and 0.56, *p* < 0.01 and <0.01, respectively).

### Phylogenetic composition of archaeal 16S rRNA assemblages

Archaeal 16S rRNA gene pyrotags were obtained from 22 of the 27 environments examined. Barcoding reactions failed for five environments (E1, E5, E6, E13) where archaeal 16S rRNA genes were detected. A total of 15692 non-chimeric sequences belonging to 31 unique OTUs (defined at 97% sequence identities) were identified in the 22 geothermal environments where sequence was obtained (Figure 5). Phylogenetic reconstruction of representatives of each unique 16S rRNA gene OTU yielded a phylogeny with resolved nodes with high bootstrap support (Figure S1 in Supplementary Material) for use in calculating Rao’s among community phylogenetic dissimilarity. The among community archaeal 16S rRNA phylogenetic dissimilarity could best be explained by variation in environmental pH (ΔAICc = 0.00, Mantel *R*^2^ = 0.06, *p* < 0.01) (Table 6). Variation in Fe^2+^ concentration was also a significant predictor of among community archaeal 16S rRNA phylogenetic dissimilarity (ΔAICc = 4.2, Mantel *R*^2^ = 0.04, *p* = 0.02). It is likely that the relationship noted between the dissimilarity in Fe^2+^ concentration and archaeal 16S rRNA gene phylogenetic dissimilarity is due to the covariance between pH and Fe^2+^ concentration in the springs examined (Table S1 in Supplementary Material).

**Table 6:** **Model ranking using ΔAICc and Mantel correlation coefficients (*R*^2^) where archaeal 16S rRNA gene Rao among community phylogenetic distance is the response variable**.

Model	ΔAICc	Mantel *R*^2^	*p*-value
pH	0.0	0.06	<0.01
Fe^2+^	4.2	0.04	0.02
${\text{NH}}_{4}^{+}$	10.0	0.02	0.17
${\text{NO}}_{3}^{-}$	11.1	0.02	0.32
Salinity	13.0	0.01	0.47
${\text{NO}}_{2}^{-}$	13.7	0.00	0.56
Temperature	13.9	0.00	0.52
S^2−^	13.9	0.00	0.67
Cl^−^	14.0	0.00	0.59
${\text{SO}}_{4}^{2+}$	14.2	0.00	0.71
Conductivity	14.3	0.00	0.72

*Explanatory variables of each model are associated with the criteria by which they were ranked*.

**Figure 4:**
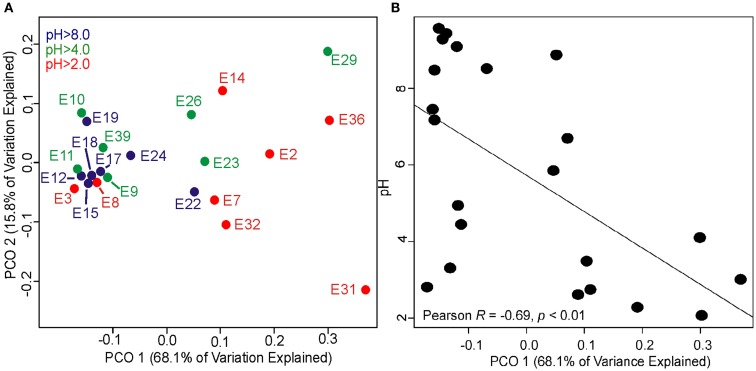
**Principle coordinates (PCO) analysis of Rao phylogenetic dissimilarity of archaeal 16S rRNA genes recovered from 22 geothermal sediments and mats with the pH of each system overlaid with color for each data point (A)**. Plot depicting relationship between PCO axis 1 coordinates and environmental pH **(B)**. A complete listing of the taxonomic composition of environments where archaeal 16S rRNA genes were recovered is presented in Table S8 in Supplementary Material. Representative sequences for each OTU are also provided in Table S8 in Supplementary Material. Regression analysis of the top five PCO axes and geochemical variables is presented in Table S9 in Supplementary Material. Regression analysis of the top five PCO axes and C- and P-iGDGTs is presented in Tables S10 and S11 in Supplementary Material, respectively.

The relatively weak relationships noted above when modeling the total variance in archaeal 16S rRNA gene Rao phylogenetic distance as a function of geochemical distance prompted a PCO analysis of archaeal 16S rRNA gene Rao phylogenetic distance to further deconvolute the complexity in the dataset. PCO ordination revealed a significant role for pH in structuring the phylogenetic composition of archaeal assemblages (Figure 4A). Assemblages sampled from acidic (pH 2.0–4.0) and alkaline (pH > 8.0) tended to cluster in the PCO ordination as a function of axis 1. PCO axis 1 (68.1% of variance explained) was significantly correlated with pH (Pearson *R* = −0.69, *p* < 0.01) (Figure 4B; Table S9 in Supplementary Material). The strong relationship noted between PCO axis 1 and environmental pH is primarily driven by the seven low pH environments that cluster in the bottom right corner of the regression analysis. Thus, acidic environments tend to harbor assemblages that are phylogenetically distinct from alkaline environments. PCO axis 2 (15.8% of variance explained) was not significantly correlated with any of the measured geochemical parameters and was most strongly correlated with temperature (Pearson *R* = 0.26, *p* = 0.25) (Table S9 in Supplementary Material).

When only considering environments where both archaeal 16S rRNA and tetraether lipids were recovered, PCO axis 1 was significantly correlated with the C-iGDGT and P-iGDGT ring indices (Pearson *R* = 0.54 and 0.49, *p* = 0.01 and 0.02, respectively) (Tables S10 and S11 in Supplementary Material), suggesting a relationship between the diversification of archaeal taxa and GDGT lipid composition. The relative abundance of a number of individual C-iGDGTs and P-iGDGTs co-varied with axes 1 and 2. The abundance of GDGT-1 and 2 in both the C- and P-iGDGT fraction were inversely correlated with PCO axis 1, consistent with the tendency for these compounds to be more abundant in alkaline environments. In contrast, the abundance of GDGT-5 and 6 in both the C- and P-iGDGT fractions were positively correlated with PCO axis 1 (Tables S10 and S11 in Supplementary Material), consistent with the tendency for these compounds to be more abundant in acidic environments. In addition, The abundance of GDGT-4′ in only the polar fraction was positively correlated with PCO axis 1. Although not significant, GDGTs with a greater number of cyclopentyl rings (e.g., GDGT-7 and 8) were inversely correlated with PCO axis 2 in both the C- and P-iGDGT fraction.

### Taxonomic composition of archaeal 16S rRNA gene assemblages

**Figure 5:**
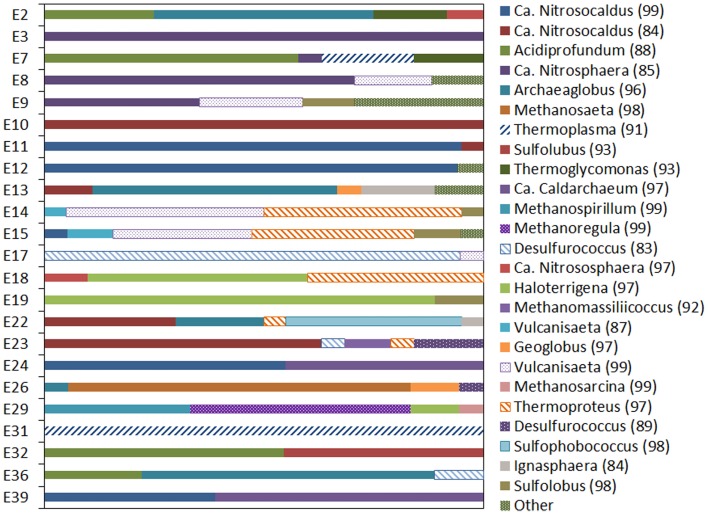
**Taxonomic composition of archaeal 16S rRNA genes**. Taxa are defined as the genus with which they were most closely affiliated based on BLASTn analysis. The percent sequence identity of each OTU to the most closely related genus is indicated in parentheses. Sequences that represented less than 0.01% of the total sequences obtained in the present study were binned as “other.” The taxonomy and environmental distribution of the genera, as depicted from top to bottom in the figure, corresponds to the OTUs, listed from top to bottom, in Table S12 in Supplementary Material.

Archaeal 16S rRNA gene OTUs were uniquely distributed as a function of environmental geochemistry. The most abundant OTU (OTU6; 30.1% of total sequences generated; Table S12 in Supplementary Material) was significantly and positively correlated with the concentration of nitrite (Pearson *R* = 0.42, *p* = 0.04) (Table S13 in Supplementary Material). OTU6 shares 99% sequence identities to the nitrifying thaumarchaeote *Candidatus* Nitrosocaldus yellowstonii and was dominant in four springs with circumneutral to alkaline pH and that had temperatures of <70°C (Figure 5; Table S12 in Supplementary Material). In addition, sequences with strong identity to *Ca*. N. yellowstonii were identified in a single acidic spring (E39; pH 4.95) with a temperature of 16.3°C. The abundance of OTU6 was also significantly and positively correlated with the abundance of archaeal *amoA* genes (Pearson *R* = 0.54, *p* < 0.01) (Table S13 in Supplementary Material), although this relationship was dependent by the high relative abundance of *amoA* in E12 (Table S5 in Supplementary Material). The second most abundant OTU (OTU51; 14.4% of total sequences generated) was identified in three circumneutral environments that ranged in temperature from 55.0 to 83.8°C (Table S13 in Supplementary Material). OTU51, which exhibited 84% sequence identity to *Ca*. N. yellowstonii (Table S12 in Supplementary Material), was not significantly correlated to any of the environmental parameters measured (Table S13 in Supplementary Material). Although not significant, the third most abundant OTU (OTU31; 10.1% of total sequences) was inversely correlated with pH (Pearson *R* = −0.36, *p* = 0.08) (Table S9 in Supplementary Material). OTU31 was distantly related to the nitrifying thaumarchaeote *Ca*. Nitrososphaera sp. EN123 (85% sequence identities) (Table S12 in Supplementary Material). The abundance of OTU42 (10.1% of total sequences generated), was significantly and inversely correlated with pH (Pearson *R* = 0.22, *p* < 0.01) (Table S13 in Supplementary Material). OTU42 exhibited 88% sequence identities to *Ca*. Aciduliprofundum boonei (Table S12 in Supplementary Material) and was dominant in four springs with acidic pH (<2.75) and had temperatures that ranged from 22.5 to 83.0°C.

### Relationship between 16S rRNA gene taxonomic composition and C- and P-iGDGT composition

A number of archaeal 16S rRNA gene OTUs co-varied with the abundances of C- and P-iGDGTs (Tables S14 and S15 in Supplementary Material respectively). For simplicity, correlations between OTU relative abundance and individual C-iGDGT lipid abundance will not be presented here given that they tended to be less significant than when the P-iGDGT fraction was considered. The relative abundance of OTU6, which is closely related to *Ca*. N. yellowstonii, was significantly correlated with the abundance of crenarchaeol (Pearson *R* = 0.56, *p* < 0.01). OTU51, which exhibited distant homology to *Ca*. N. yellowstonii was not significantly correlated with the abundance of crenarchaeol or any other individual P-iGDGTs. The abundance of OTU31, which is distantly affiliated with *Ca*. Nitrosphaera sp. EN123 and which was detected in acidic springs, was significantly correlated with the abundances of P-iGDGTs with seven or more rings.

## Discussion

The ability for microbial populations to radiate into a new ecological niche is dependent on physiological compatibility with the physical and chemical characteristics of the local environment (Kassen, 2009; Losos, 2010), due in large part to the additional energetic demands that physical and chemical stress impose on cells (Hoehler, 2007; Valentine, 2007). Cellular membranes and the electrochemical potential that is generated across them are critical components in the production of energy for use in biomolecular synthesis and maintenance (van de Vossenberg et al., 1998; Macalady et al., 2004; Baker-Austin and Dopson, 2007). Thus, maintaining a stable and functional lipid membrane is fundamental to energy transduction and the diversification of life into new ecological niches. Here, we quantitatively integrate biomolecular lipid, genetic, and environmental data in YNP geothermal environments in order to better define the role of geochemical gradients in driving the taxonomic and phenotypic diversification of archaea.

Gradients in geothermal fluid pH were shown to influence the degree of C- and P-iGDGT cyclization (RI), with acidic fluids tending to select for lipids with more internal cyclopentyl rings. These observations are consistent with previous characterizations of C-iGDGT lipid composition in geothermal habitats, including those in YNP (Schouten et al., 2007; Pearson et al., 2008). As shown here, the degree of cyclization of P-iGDGTs is inversely correlated with pH in geothermal habitats. Given that P-iGDGTs are purported to better reflect living or intact cells when compared to C-iGDGTs (Sturt et al., 2004; Biddle et al., 2006; Schubotz et al., 2009), these results indicate that the active fraction of archaeal assemblages is responding to gradients in pH and this signature is preserved in the C-iGDGT fraction.

Experimental and molecular modeling studies indicate that tetraether lipids and the extent of internal tetraether lipid cyclization confer resistance to proton permeation. For example, studies of archaeal liposomes formed from tetraether lipids derived from the archaean *S. acidocaldarius* exhibit a much lower proton permeation over a temperature range of 20–80°C when compared to bacterial acyl-ester lipids (Elferink et al., 1994). Molecular modeling of these liposomes suggest the decreased proton permeability relative to acyl membranes results from both the ether bonding and the bulky isoprenoid lipid core (Yamauchi et al., 1993; Komatsu and Chong, 1998; Mathai et al., 2001). In addition, hydrogen bonding between the sugar residues exposed at the face of the lipid monolayer has been proposed to reduce proton permeability, although to a lesser extent than the rigid and dense packing of the isoprenoid core (Komatsu and Chong, 1998). Additional studies are clearly warranted to investigate the influence of GDGT lipid sugar head groups in geothermal environments, and their relation to taxonomic evolution. In separate molecular simulations of archaeal liposomes, it was found that tetraether lipid isoprenoid cyclization enhances the resistance to proton permeation through increased packing density (Gabriel and Chong, 2000). The importance of cyclopentyl rings in resistance to proton permeation, as suggested by the environmental data presented here, is supported by a survey of the GDGT lipid composition of archaeal isolates which indicates that acidophiles, when cultivated at their optimum pH and temperature, synthesize GDGT lipids containing a greater average number of cyclopentyl rings than neutrophiles (Macalady et al., 2004). Moreover, these findings are consistent with recent experimental data that indicate that the thermoacidophile *A. sulfurireducens* increase the degree of GDGT lipid cyclization in response to increasing acidity (Boyd et al., 2011b).

Gradients in geothermal fluid pH select for distinct lineages of archaea, with acidic (pH < 4.0) environments tending to be dominated by sequences affiliated with *Vulcanisaeta* sp., *Thermoplasma* sp., *Sulfolobus* sp., and *Thermoproteus* sp. The recovery of sequences affiliated with these taxa is consistent with the thermoacidophilic nature of the type strains (Darland et al., 1970; Brock et al., 1972; Zillig et al., 1981; Itoh et al., 2002), many of which were originally isolated from YNP. In addition, a number of studies have previously identified sequences affiliated with these taxa in acidic geothermal springs in YNP (Inskeep et al., 2005; Meyer-Dombard et al., 2005; Spear et al., 2005; Kozubal et al., 2008; Reno et al., 2009; Macur et al., 2013). The lipid membranes of representatives of these lineages are known to be comprised primarily of tetraether lipids, suggesting this as a key to survival in high temperature and/or acidic environments (De Rosa and Gambacorta, 1988; Macalady et al., 2004).

PCO analysis indicates that the phylogenetic relationships among archaeal assemblages is driven primarily by pH gradients, with significant correlations noted between assemblage clustering and the C- and P-iGDGT ring indices. This form of data integration directly links the composition of tetraether lipids with the diversification of archaeal lineages. Other factors are invariably involved in driving the diversification of archaeal lineages such as differences in the energetic favorability of redox couples as a function of spring pH (Inskeep et al., 2005; Meyer-Dombard et al., 2005; Shock et al., 2010), functional adaptation of lineages to utilize those redox gradients (Inskeep et al., 2010), and the evolution of strategies aimed at detoxifying metals that tend to be more soluble and/or bioavailable under acidic conditions (e.g., mercury; Wang et al., 2011), among others. Nonetheless, the fundamental requirement for cells to maintain functional lipid membranes in order to preserve electrochemical gradients and cellular homeostasis (van de Vossenberg et al., 1998; Macalady et al., 2004; Baker-Austin and Dopson, 2007) is expected to represent a first order constraint on the ability for populations to diversify into new pH realms.

A number of C- and P-iGDGT lipids were found to co-vary with the abundance of 16S rRNA gene sequences affiliated with taxa thought to be involved in their synthesis, most notably crenarchaeol and *Ca*. N. yellowstonii. *Ca*. N. yellowstonii is an obligate nitrifying autotroph that has been demonstrated to synthesize crenarchaeol (de la Torre et al., 2008). Sequences closely affiliated with *Ca*. N. yellowstonii were recovered from several circumneutral to alkaline geothermal springs in YNP both in this study, and in previous studies (de la Torre et al., 2008). The abundance of sequences affiliated with *Ca*. N. yellowstonii, as well as the abundance of P-crenarchaeol, were inversely correlated with the concentration of ${\text{NH}}_{4}^{+}$ and significantly and positively correlated with the concentration of ${\text{NO}}_{2}^{-}$, a product of nitrification. These data may suggest that populations of *Ca*. N. yellowstonii are involved in nitrification in the environments analyzed. In support of this conclusion, the abundance of 16S rRNA gene sequences closely affiliated with *Ca*. N. yellowstonii was significantly and positively correlated with the abundance of archaeal *amoA* sequences, the product of which is required to oxidize ${\text{NH}}_{4}^{+}$ to the intermediate hydroxylamine. However, not all geothermal mat/sediment samples that exhibited elevated abundances of archaeal *amoA* sequences (>10^3^ copies/ng DNA) yielded 16S rRNA gene sequences closely affiliated with *Ca*. N. yellowstonii (e.g., E3, E7, E16–E19). In some instances (e.g., E3, E7), this discrepancy might be explained by the detection of 16S rRNA gene sequences closely affiliated with the soil nitrifier *Ca*. Nitrososphaera sp. which also encodes for *amoA* (Tourna et al., 2011). Geothermal springs E3 and E7 are moderate temperature and acidic environments and while previous studies have shown evidence for nitrification activity in acidic geothermal springs (Reigstad et al., 2008), it is unclear if the activity could be attributed to populations of *Ca*. Nitrososphaera spp. The abundance of crenarchaeol in YNP geothermal environments in both the C- and P-iGDGT fractions was, however, closely correlated with the abundance of archaeal *amoA*, implying a link between nitrification and crenarchaeol production. In accordance, the abundance of crenarchaeol in the C- and P-iGDGT fractions was inversely associated with the concentration of ${\text{NH}}_{4}^{+}$ and positively associated with the abundance of ${\text{NO}}_{2}^{-}$.

It has been suggested that the previous detection of crenarchaeol in geothermal springs in the GB, Nevada (Zhang et al., 2006), which host abundant populations of nitrifying archaea (Costa et al., 2009; Dodsworth et al., 2011), is the result of allochthonous input of lipid material from the weathering of nearby soils (Schouten et al., 2007). Our pairwise comparison of geothermal mat/sediment C- and P-iGDGT profiles with those from surrounding soils, which reveals no association between the two, strongly suggests that the GDGTs detected in geothermal springs is unlikely to be solely the result of allochthonous input from surrounding environments. This conclusion is supported by the recent demonstration of P-crenarchaeol production in two geothermal springs in the GB (Pitcher et al., 2009). However, given the results noted above, namely the lack of sequences affiliated with known nitrifiers in springs where crenarchaeol is detected, limited input of allochthonous material from surrounding environments cannot be excluded.

## Conclusion

In summary, the results presented here quantitatively demonstrate links between environmental variation, the composition of archaeal GDGT lipids, and the distribution of archaeal lineages in geothermal environments. The detection of P-iGDGT lipids in ecosystems that range in pH from 2.06 to 9.57 and temperature from 16.3 to 86.7°C indicates that archaea have diversified to inhabit a wide range of environments, implying a fundamental role for GDGT lipids in this process. The relationships presented here are strongly dependent on the inclusion of acidic ecosystems, suggesting that ability to adjust GDGT lipid composition is a critical phenotype that enabled the diversification of thermophilic archaea into, or out of, acidic habitats. The close association noted between the distribution of 16S rRNA genes closely affiliated with characterized nitrifying archaea, archaeal *amoA* gene sequences, and the presence of crenarchaeol support the hypothesis that this lipid is a biomarker for thaumarchaeotes involved in nitrification activity.

## Conflict of Interest Statement

The authors declare that the research was conducted in the absence of any commercial or financial relationships that could be construed as a potential conflict of interest.

## Supplementary Material

The Supplementary Material for this article can be found online at http://www.frontiersin.org/Terrestrial_Microbiology/10.3389/fmicb.2013.00062/abstract

## Footnotes

^1^http://pagesperso-orange.fr/giraudoux/

^2^http://vegan.r-forge.r-project.org/

^3^http://ecology.msu.montana.edu/labdsv/R
